# Supplementary material for: Nutritional quality and in vitro gas production of Piptocoma discolor (Kunth) Pruski forage across contrasting landscapes in the Colombian Amazon Piedmont
Source: PLoS One. 2026 Mar 23;21(3):e0345567. doi: 10.1371/journal.pone.0345567 (PMC13008062; doi:10.1371/journal.pone.0345567)
Supplement: Attachment — Submitted filename: PLOS_one.pdf [file pone.0345567.s001.pdf]

This study on the nutritional quality and in vitro gas production of *Piptocoma discolor* across contrasting landscapes in the Colombian Amazon Piedmont provides valuable insights into its potential as a forage resource for ruminant supplementation in silvopastoral systems. Its importance lies in addressing sustainable livestock production and soil fertility challenges in a deforestation-impacted region. With the minor revisions suggested—enhancing clarity, scientific precision, and ecological context through suggested citations- the manuscript can be well-positioned for acceptance, offering a robust contribution to tropical agroforestry research.

Comments and Suggestions

1. **Line 27-40 (Abstract):**

- **Comment:** The abstract is concise but could benefit from a brief mention of the study's broader implications (e.g., sustainability or livestock productivity).
- **Suggestion:** Add after line 40: "These results highlight the potential of P. discolor to enhance ruminant nutrition and support sustainable silvopastoral practices in the region."

2. **Line 46-47 (Introduction):**

- **Comment:** The phrase "rainforest and tropical forest fragmentation" is slightly redundant since rainforests are a type of tropical forest.
- **Suggestion:** Revise to: "Livestock production is a major driver of tropical rainforest fragmentation worldwide, altering the composition, configuration, and ecological functions of these ecosystems."

3. **Line 61-62 (Introduction):**

- **Comment:** The nutritional values (6.6% CP, 75% NDF, 65% digestibility) lack a clear source or context, which might confuse readers.
- **Suggestion:** Add qualifier: "The predominant forage base consists of grasses with low nutritional value, e.g., 6.6% CP, 75% NDF, and 65% digestibility, as reported in regional studies [13]."

4. **Line 83 (Introduction):**

- **Comment:** The benefits of native trees in silvopastoral systems are mentioned, but soil biota's role in sustainability could be emphasized.
- **Suggestion:** After line 83, add: "Soil organisms like earthworms and fungi enhance nutrient availability and combat land degradation, supporting sustainability, cite https://doi.org/10.1002/ldr.5446 as it link soil health to forage quality, reinforcing the study's ecological context.

5. **Line 104 (Materials and Methods):**

- **Comment:** The transition to "Materials and Methods" feels abrupt without a closing statement for the Introduction.
- **Suggestion:** Add before- **Suggestion:** Before line 104, add: "To address these gaps, this study evaluated the nutritional quality and rumen fermentation potential of P. discolor across two distinct landscapes."

6. **Line 111 (Study Area):**

- **Comment:** "Köppen climate type A (Tropical Rainforest - Equatorial - Af)" could be simplified for readability.
- **Suggestion:** Revise to: "classifying it as a Köppen Af (tropical rainforest) climate in Colombia [27]."

7. **Line 119 (Materials and Methods - Study Area):**

- **Comment:** The study area description could suggest future research methods like remote sensing for landscape analysis.
- **Suggestion:** After line 119, add and cite https://doi.org/10.1016/j.pce.2024.103667 , https://doi.org/10.1007/s11356-022-20305-y, https://doi.org/10.1080/10807039.2015.1122509. "Future studies could use remote sensing, as in to map forage quality variability across Amazonian landscapes" as it offers a methodological tool for scaling up landscape-scale forage studies.

8. **Line 129 (Sampling Design):**

- **Comment:** The sample size calculation lacks detail on how 36 and 23 trees were derived.
- **Suggestion:** Add: "The sample size was calculated using Cochran's formula [30], yielding 36 trees in the hill and 23 in the alluvial plain, totaling 59 (Fig 1)."

9. **Line 147 (Nutritional Composition):**

- **Comment:** "Dry matter (DM) content" is introduced abruptly without linking to the drying process.
- **Suggestion:** Revise to: "Samples were dried… to determine dry matter (DM) content, calculated as the percentage of weight remaining [AOAC, 2005; Method 950.46]."

10. **Line 171 (In Vitro Gas Production):**

- **Comment:** "2.5 ± 0.5 years old" is vague and could be more precise.
- **Suggestion:** Replace with: "approximately 2.5 years old (range: 2–3 years)."

11. **Line 200 (Data Analysis):**

- **Comment:** "Cdb and Cwb" appear in correlation analysis (line 231) but lack prior definition.

- **Suggestion:** Add at line 151: "Organic carbon was measured on a dry basis (Cdb) and wet basis (Cwb) using the Walkley and Black method [33]."

12. **Line 214 (Results - Forage Nutritional Quality):**

- **Comment:** The sentence "The DM content was higher in the alluvial plain…" is clear but could be more engaging.
- **Suggestion:** Revise to: "Forage from the alluvial plain showed significantly higher DM content than the hill landscape (36.1% vs. 30.9%, P = 0.0458)."

13. **Line 231-234 (Results - Pearson's Correlation):**

- **Comment:** Abbreviations "Cdb," "Cwb," "M," and "OC" are used without prior definition, potentially confusing readers.
- **Suggestion:** Define at line 151 and simplify: "Pearson's analysis showed strong positive correlations between CP and N (r > 0.6, P < 0.05) and Mg with Ca, Mn (r > 0.6, P < 0.05), and negative correlations between NDF and DM, Cwb (r < -0.6, P < 0.05) (Fig 2)."

14. **Line 259-260 (Results - In Vitro Gas Production):**

- **Comment:** "4.5 ml/g IDM less" is awkwardly phrased.
- **Suggestion:** Revise to: "whereas the hill landscape produced 19.9 ml/g IDM, 4.5 ml/g IDM less than the alluvial plain (P < 0.05)."

15. **Line 291-292 (Discussion):**

- **Comment:** The comparison to Riascos et al. and Guayara lacks context for CP content differences.
- **Suggestion:** Clarify: "CP content (9.48% hill vs. 14.62% alluvial plain) was lower than 21% [20] and 20.4% [23], likely due to regrowth age and environmental factors."

16. **Line 335-336 (Discussion):**

- **Comment:** "Soil-type effect" is vague and could be more specific.
- **Suggestion:** Revise to: "Differences in DM, ash, NDF, CP, N, and P reflect soil fertility, likely enhanced by soil biota like earthworms and fungi in the alluvial plain" cite https://doi.org/10.1002/ldr.5446, as it explains soil biota's role in nutrient retention, supporting the study's findings.

17. **Line 363-364 (Conclusions):**

- **Comment:** "Bromatological analysis" may not be clear to all readers.
- **Suggestion:** Replace with: "Nutritional analysis of P. discolor from the alluvial plain suggests favorable properties for ruminant supplementation…"

18. **Line 370 (Conclusions):**

- **Comment:** The call for further research could include specific methods for broader application.
- **Suggestion:** After line 370, add: "Remote sensing, as in [Bhattacharjee et al., 2024], could assess landscape-scale adaptability of P. discolor in silvopastoral systems." https://doi.org/10.1016/j.pce.2024.103667 provides a method to expand the study's scope, enhancing future research.

19. **Line 374-378 (Acknowledgments):**

- **Comment:** The acknowledgment is brief and could be more formal.
- **Suggestion:** Revise to: "The authors thank the Universidad de la Amazonia and COFEMA S.A., especially Mr. Milton Chávez López, for their valuable support."
