# Supplementary material for: Nutritional quality and in vitro gas production of Piptocoma discolor (Kunth) Pruski forage across contrasting landscapes in the Colombian Amazon Piedmont
Source: PLoS One. 2026 Mar 23;21(3):e0345567. doi: 10.1371/journal.pone.0345567 (PMC13008062; doi:10.1371/journal.pone.0345567)
Supplement: Attachment — Submitted filename: Response to reviewers.docx [file pone.0345567.s003.docx]

Florencia, Colombia, November 26, 2025

Editors-in-Chief

*PLOS ONE*

Dear Editors:

We attach a revised version of our manuscript: “Nutritional quality and in vitro gas production of forage Piptocoma discolor (Kunth) Pruski in contrasting landscapes of the Colombian Amazonian piedmont”.

We sincerely thank the editor for his positive and encouraging evaluation and advice to improve the manuscript before acceptance for publication. All comments were reasonable and were taken into account when revising our manuscript. We hope that this revised version of the manuscript has improved with the modifications and can now be considered for publication.

**EDITOR**

**Additional requirements.**

Please respond to the following query regarding the map contained in Figure 1.

- Where did the authors obtain the maps, basemaps, shapefiles, map data, etc in this Figure?

**Response 1:**

The maps, basemaps, shapefiles, and all other cartographic inputs used in the figure were obtained from the Open Data portal of the Agustín Codazzi Geographic Institute (IGAC). All geographic layers—including municipal and departmental boundaries, drainage networks, roads, and other spatial elements—originate exclusively from this official platform, which is publicly available for download and free use under its open data license.

- What software was used to make these maps?

**Response 2:**

The map was produced using QGIS, an open-source geographic information system that enables the processing, analysis, and visualization of spatial data. All cartographic layers obtained from IGAC’s Open Data repository were integrated, processed, and rendered within this software environment to generate the final figure.
