# Supplementary material for: Nutritional quality and in vitro gas production of Piptocoma discolor (Kunth) Pruski forage across contrasting landscapes in the Colombian Amazon Piedmont
Source: PLoS One. 2026 Mar 23;21(3):e0345567. doi: 10.1371/journal.pone.0345567 (PMC13008062; doi:10.1371/journal.pone.0345567)
Supplement: Attachment — Submitted filename: Response to reviewers..docx [file pone.0345567.s004.docx]

Florencia, Colombia, March 3, 2026

Editors-in-Chief

*PLOS ONE*

Dear Editors:

We attach a revised version of our manuscript: “Nutritional quality and in vitro gas production of forage Piptocoma discolor (Kunth) Pruski in contrasting landscapes of the Colombian Amazonian piedmont”.

We sincerely thank the editor for his positive and encouraging evaluation and advice to improve the manuscript before acceptance for publication. All comments were reasonable and were taken into account when revising our manuscript. We hope that this revised version of the manuscript has improved with the modifications and can now be considered for publication.

**EDITOR**

**Additional requirements.**

1. If the authors have adequately addressed your comments raised in a previous round of review and you feel that this manuscript is now acceptable for publication, you may indicate that here to bypass the “Comments to the Author” section, enter your conflict of interest statement in the “Confidential to Editor” section, and submit your "Accept" recommendation.

Reviewer #1: (No Response)

Reviewer #2: All comments have been addressed

**Response:** All comments mentioned by the reviewers have been taken into account for the improvement of the article, its submission, and its acceptance.

**2.**  Review Comments to the Author Please use the space provided to explain your answers to the questions above. You may also include additional comments for the author, including concerns about dual publication, research ethics, or publication ethics. (Please upload your review as an attachment if it exceeds 20,000 characters)

Reviewer #1: **The manuscript is scientifically sound and significantly improved. If the authors confirm the inclusion of the minimal raw data set and add the specific map attribution, it is ready for publication .**

Reviewer #2: well, done! no additional comments from my side, all the points were properly addressed by the authors

**Response:** Given the rejection of the map's inclusion in the manuscript, the decision was made to remove it, as a previous version had been sent with justification and corrections but was rejected. Therefore, the map is no longer part of the manuscript. The responses sent to the journal editor for review are listed below, but they were not accepted.

**The maps, basemaps, shapefiles, and all other cartographic inputs used in the figure were obtained from the Open Data portal of the Agustín Codazzi Geographic Institute (IGAC). All geographic layers—including municipal and departmental boundaries, drainage networks, roads, and other spatial elements—originate exclusively from this official platform, which is publicly available for download and free use under its open data license.**

**The map was produced using QGIS, an open-source geographic information system that enables the processing, analysis, and visualization of spatial data. All cartographic layers obtained from IGAC’s Open Data repository were integrated, processed, and rendered within this software environment to generate the final figure.**
